# 

**Published:** 1892-04

**Authors:** 


### Society Meetings.

The American Medical Association will hold its forty-third annual session, in Detroit, on Tuesday, Wednesday, Thursday, and Friday, June 7, 8, 9, and 10, 1892. Dr. Henry O. Marcy, of Boston, is president, and Dr. Henry O. Walker, of Detroit, is chairman of the Committee of Arrangements. The general addresses will be delivered by the following named gentlemen: On General Medicine, Dr. J. S. Cain, of Tennessee; on General Surgery, Dr. John B. Hamilton, of Illinois; on State Medicine, by Dr. C. A. Lindsley, of Connecticut. It is anticipated that this will be one of the most successful meetings of the Association, and the local committee in Detroit is making rapid progress in perfecting arrangements to care for a large gathering.

Dr. E. E. Montgomery, chairman of the section of Obstetrics and Diseases of Women,—address, 1818 Arch street, Philadelphia,—would be glad to receive the titles of proposed papers from those who desire to take part in the proceedings of this section at the next meeting of the Association. It is important that titles should be presented as early as possible, in order to make proper arrangements for their careful consideration and discussion.

The first Pan-American Medical Congress will meet in Washington, D. C., on Tuesday, Wednesday, Thursday, and Friday,

September 5, 6, 7, and 8, 1893. The Congress will be presided over by Dr. William Pepper, President, of Philadelphia. The Secretary-General, Dr. Charles A. L. Reed, of Cincinnati, has displayed rare executive capacity in so completely putting the machinery of the Congress in working order as to already assure its success. Dr. A. M. Owen, of Evansville, Ind., is Treasurer, and it is desirable that all the officers of the Congress, and as many as possible of those who desire to take part therein, pay in the membership fee of $10.00 at an early date.

---

An International Periodical Congress of Gynecology and Obstetrics has been called, and its first session will be held in Brussels, Belgium, September 14 to 19, 1892. Dr. Jacobs, of 12 Rue des Petits-Carmes, Brussels, is the Secretary-General, and Dr. F. Henrotin, 353 La Salle avenue, Chicago, is the American Secretary, and all communications from this country should be addressed to the latter.

Mr. Lawson Tait will preside at the Congress, and the leading questions offered for discussion are as follows:

1. Pelvic Suppuration, referee, Dr. Paul Segond, Paris.
2. Extrauterine Pregnancy, referee, Dr. A. Martin, Berlin.
3. Placenta Previa, referee, Dr. Berry Hart, Edinburgh.

The indications are that this will be a large meeting of the leading obstetricians and gynecologists in the world.

Notice to Contributors.—We are glad to receive contributions from every one who knows anything of interest to the profession. Articles designed for publication in the Journal should be handed in before the first day of the month. The Editors are not responsible for the views or opinions of contributors. All communications should be addressed to the Managing Editor, 284 Franklin St., Buffalo, N. Y.

